# Supplementary material for: Clinical Prediction Models for Prognosis of Colorectal Liver Metastases: A Comprehensive Review of Regression-Based and Machine Learning Models
Source: Cancers (Basel). 2024 Apr 25;16(9):1645. doi: 10.3390/cancers16091645 (PMC11083016; doi:10.3390/cancers16091645)
Supplement: Supplementary file 1 [file cancers-16-01645-s001.zip › cancers-2977917-supplementary.pdf]

**Supplementary methods:** Details of the search strategy

(Pubmed)

#1. ((colorectal liver metastas*[Title/Abstract]) OR (CRLM[Title/Abstract]))

#2. ((machine learning[Title/Abstract]) OR (neural network*[Title/Abstract]) OR (prediction model*[Title/Abstract]) OR (clinical score*[Title/Abstract]) OR (prognostic score*[Title/Abstract]) OR (risk score*[Title/Abstract]) OR (prediction score*[Title/Abstract]) OR (prognostic model*[Title/Abstract]) OR (validat*[Title/Abstract]) OR (calibrat*[Title/Abstract]))

#3. #1 AND #2

**Table S1:** Characteristics of traditional clinical prediction models for prognosis of colorectal liver metastases

| First Author | Year of publication | Country | Study period | Study design | Multicentre | Model type | Univariate screening of predictors | Predictors | Outcome | Patient number | Internal/External validation | Missing data |
|---|---|---|---|---|---|---|---|---|---|---|---|---|
| **Nordlinger** | 1996 | France | 1968-1990 | retrospective | yes | Cox regression | yes | • Age<br>• Extension into serosa of the primary cancer (T stage)<br>• Lymphatic spread of the primary cancer (N stage)<br>• Time interval from primary tumor to metastases<br>• Size of largest metastases<br>• Number of metastases<br>• Clearance | OS | 1568 | Bootstrapping | No information |
| **Fong** | 1999 | USA | 1985-1998 | retrospective | no | Cox regression | yes | • Nodal status of primary (N stage)<br>• disease-free interval from the primary to discovery of the liver metastases of <12 months<br>• number of tumors >1<br>• preoperative CEA level >200 ng/ml<br>• size of the largest tumor >5 cm | DFS | 1001 | Not performed | No information |

| | | | | | | | | | | | | |
|---|---|---|---|---|---|---|---|---|---|---|---|---|
| **Iwatsuki** | 1999 | USA | 1981-1996 | retrospective | no | Cox regression | yes | • Liver metastases>2<br>• Metastasis size >8 cm<br>• Bilobar distribution<br>• Interval between colorectal and liver resection <30 months<br>• Margin involved<br>• Extrahepatic disease | OS and DFS | 305 | Not performed | No information |
| **Lise** | 2001 | Italy | 1977-1997 | retrospective | no | Cox regression | yes | • >30% liver invasion<br>• Node-positive primary<br>• Liver metastases >1<br>• GPT levels >55 U/l<br>• Non-anatomical resection | DFS | 135 | Not performed | No information |
| **Nagashima** | 2004 | Japan | 1981-2001 | retrospective | yes | Cox regression | yes | • serosal invasion of primary<br>• lymph node positive primary<br>• Number of hepatic metastatic tumors<br>• Diameter of hepatic metastatic tumor >5cm<br>• extrahepatic metastasis | CSS | 81 (dev), 70 (val) | External validation cohort | No information |
| **Schindl** | 2005 | UK, Austria | 1988-2002 | retrospective | yes | Cox regression | yes | • Duke stage<br>• number of metastases >3<br>• ALP<br>• CEA<br>• Albumin | OS | 269 (dev), 193 (val) | External validation cohort | No information |
| **Malik** | 2007 | UK | 1993-2006 | retrospective | no | Cox regression | yes | • Inflammatory response to tumour<br>• Liver metastases >8 | OS and DFS | 687 | Not performed | Complete case analysis |

| | | | | | | | | | | | | |
|---|---|---|---|---|---|---|---|---|---|---|---|---|
| **Ueno** | 2000 | Japan | 1985-1996 | retrospective | no | Cox regression | yes | • marked tumour budding or node-positive primary<br>• Liver metastasis <12m<br>• Liver metastases >3 | OS and Recurrence | 85 | Not performed | No information |
| **Lee** | 2007 | South Korea | 1994-2005 | retrospective | no | Cox regression | yes | • Margin <5 mm<br>• CEA >5 ng/ml<br>• Node-positive primary >4<br>• Liver metastases >1 | OS | 135 | Not performed | No information |
| **Konopke** | 2009 | Germany | 1993-2006 | retrospective | no | Cox regression | yes | • CEA level >200 ng/ml<br>• number of liver metastases>4<br>• time of diagnosis of the liver metastases synchronous to primary colorectal carcinoma | Mortality and recurrence | 201 | Bootstrapping | No information |
| **Tanaka** | 2004 | Japan | 1992-2001 | retrospective | no | Cox regression | yes | • Histology of primary lesion (poor/muc)<br>• Vascular invasion (marked)<br>• Distribution of metastases (bilobar)<br>• doubling time of metastatic lesions (45 days) | OS and PFS | 149 | Not performed | No information |
| **Minagawa** | 2007 | Japan | 1980-2002 | retrospective | yes | Cox regression | yes | • Hepatic LN mets<br>• Number of LN mets around primary cancer<br>• CEA<br>• Number of LM | OS | 369 (dev), 229 (val) | External validation | No information |

| | | | | | | | | | | | | |
|---|---|---|---|---|---|---|---|---|---|---|---|---|
| **Yamaguchi** | 2008 | Japan | 1992-1996 | retrospective | yes | Cox regression | yes | • number of liver metastases<br>• size of the largest liver metastases<br>• mesenteric lymph node metastases<br>• extrahepatic metastases | OS | 478 | Not performed | Complete case analysis |
| **Adam** | 2011 | France | 1990-2006 | retrospective | no | Cox regression | yes | • primary tumor located in the right colon<br>• EHD concomitant to CLM recurrence<br>• at least 6 CLM at diagnosis<br>• preoperative CEA level >10 ng/mL<br>• absence of isolated lung metastases | OS | 186 | Not performed | No informatio n |

OS: Overall survival, CEA: Carcinoembryonic antigen, DFS: Disease-free survival, GPT: serum glutamic pyruvic transaminase, dev: development, val: validation, ALP: Alkaline phosphatase, CSS: Cancer-specific survival, muc: mucinous, LN: Lymph node, EHD: Extrahepatic disease, CLM: Colorectal metastases

**Table S2:** Inclusion/Exclusion criteria and median follow-up of studies developing clinical prediction models for prognosis of colorectal liver metastases

| First Author (year) | Inclusion criteria | Exclusion criteria | Median follow-up (months) |
|---|---|---|---|
| **Buisman (2022)** | Patients after curative resection and/or ablation of CRLM | Incomplete resection, residual extrahepatic disease, no colorectal resection | 99 (IQR 53–160) |
| **Bertsimas (2022)** | Patients who underwent curative intent surgery for CRLM and had known KRAS status | Patients with missing data, incomplete follow-up, patients with margin width >20mm | NR |
| **Bao (2021)** | NGS data available | - | NR |
| **Lam (2023)** | Patients who underwent hepatectomy for CRLM | Unresected primary colorectal malignancy before/during the entire study period, first liver metastasis treated by RFA only, postoperative mortality, and multiple synchronous primary malignancies. | 43.2 (IQR 24–68.4) |
| **Reijonen (2023)** | Liver resection with curative intent for CRLM | - | NR |
| **Margonis (2018)** | Patients that underwent complete resection of CRLM and had available data on KRAS status | Patients who only underwent ablation or palliative liver resection (R2 resection) | 30.5 (IQR 15.7–55.1) |
| **Paredes (2020)** | Patients with primary CRC and liver metastases | Patients who underwent palliative resection | 26.9 (range 0.1–200.2) |
| **Fruhling (2021)** | Patients who underwent surgery with curative intent for CRLMs | - | 108 (IQR 103–112) |
| **Taghavi (2021)** | Histopathology confirmed colorectal adenocarcinoma, primary cM0 stage, no evidence of extrahepatic metastases on primary or follow-up imaging, and availability of a primary staging CT including a portal venous phase before the start of any treatment. | Previous liver surgery, previous liver disease such as steatosis or cirrhosis, and previous systemic chemotherapy before diagnosis of the colorectal tumor. | 1 of 2 groups: 51 (range 24–70) |
| **Brudvik (2019)** | Patients undergoing resection of CRLM with known RAS mutation status and known values for all 5 t-CS factors | If concomitant radiofrequency ablation was used or if the resection was considered noncurative before surgery | NR |

| | | | |
|---|---|---|---|
| **Moaven (2023)** | CRLM patients who underwent surgical resection with curative intent and had available information in electronic medical records | Patients who underwent ablation only | 32 |
| **Villard (2022)** | Occurrence of CRLM discussed at MDT conference | Lack of data on survival and follow-up | 39.6 |
| **Chen (2020)** | Patients who underwent curative-intent liver resection for CRLM and had available data on KRAS/NRAS/BRAF mutation status | The histologic type of tumor was not called adenocarcinoma, peritoneal metastasis was present, R2 resection, history of previous hepatectomy, and incomplete data. | NR |
| **Chen (2022)** | Pathologically confirmed CRLM; considered a resectable disease by a multidisciplinary team (MDT) before surgery, no evidence of extrahepatic disease other than lung metastases, complete resection of the metastases and primary site. | - | 42 |
| **Dai (2021)** | CRC patients with synchronous or metachronous liver metastases, patients who underwent single-stage or two-stage surgeries with curative intent, adenocarcinoma as the pathological type, with or without neoadjuvant chemotherapy. | Recurrent CRLM, remnant lesions confirmed by post-operative radiological/ultrasound ex-amination, noncompliance with routine post-operative surveil-lance, lost to follow-up, incomplete medical record. | NR |
| **Liu (2021)** | First recurrence developed after initial radical resection of both the liver metastasis and primary tumor, the initial recurrence was considered resectable and liver-limited by a multidisciplinary team (MDT), no extrahepatic metastasis had occurred since the first hepatic resection; and there were no other simultaneous malignancies. | - | 39 (95% CI: 35–43) |
| **Liang (2021)** | Pathologically diagnosed CRLM, both the primary lesions and hepatic metastases underwent radical surgery with tumor-negative resection margins (R0) and post-hepatectomy recurrence detected before June 2019 | Patients without active follow-up or adequate clinical information for analysis, patients with a history of other malignancies. | Training: 27.7, Validation: 26.6 |

| | | | |
|---|---|---|---|
| **Wu (2021)** | Initially diagnosed as having synchronous CRLM, Eastern Cooperative Oncology Group status ≤2, indicating no surgical contradictions and with simultaneous resection of primary tumor and liver metastasis pathologically confirmed malignant primary tumor and liver metastasis, available and complete clinical records, including pathologic diagnosis, treatment strategy, and follow-up. | Patients who had either malignant tumors in other organs, or palliative resection of tumors. | NR |
| **Sasaki (2022)** | Curative-intent hepatic resection of CRLM | - | 39.7 |
| **Huiskens (2019)** | Patients who underwent ALPPS for suspected CRLM | Patients with diagnoses other than CRLM, such as patients without a reported 90-day survival status or without reported details on morbidity | NR |
| **Bai (2022)** | Histologically confirmed CRC, evaluated as having resectable CRLM at initial diagnosis or after preoperative conversion therapy by a multidisciplinary team (MDT), R0 resection of CRLM, had blood biochemical examination data within 1 month before hepatectomy and were postoperatively followed up for at least 3 months. | Previous history of hepatectomy, presence of peritoneal metastasis, ablation of metastatic sites or transcatheter hepatic arterial chemoembolization (TACE) within 1 month of hepatectomy, incomplete medical records and previous history of a malignant tumor | 62.9 |
| **Fang (2022)** | Over 18 years old, diagnosed with CRLM by medical imaging examination or postoperative pathological examination, no other evidence of distant metastasis, complete data on ApoA-I and NLR | Other primary tumors or combined with other serious diseases, other diseases associated with blood lipid levels (such as diabetes, hyperlipidemia, or metabolic syndrome), receiving hormonal medication or taking any drugs that inhibit lipid metabolism, absence of follow-up records | NR |

| | | | |
|---|---|---|---|
| **Qin (2022)** | Patients who underwent US-guided percutaneous MWA for CRLM, patients with CRC confirmed by a pathology study, patients with liver-limited metastases and no major vascular incursion, number of CRLM <9 and size of CRLMs <5 cm. | Child-Pugh class C or severe coagulation disorder, poor performance status (ECOG PS>2), no surgical removal of the primary tumor, inability to detect the lesions on US or contrast-enhanced ultrasound(CEUS), inability to completely ablate the lesions because of proximity to major biliary structures or adherence to the gastrointestinal tract, inability for the patient to tolerate the pain during the procedure and termination of the treatment, insufficient follow-up time | 23.5 (range, 6.0–74.2) |
| **Kawaguchi (2021)** | Onitial liver resection of CRLM with curative intent | Patients with<br>unknown RAS mutation status | 66 (95 % CI: 62.4-69.6) |
| **Zhang (2023)** | Availability of DEGs | - | NR |
| **Chen (2021)** | Histologically proven colorectal adenocarcinoma liver metastases, patients underwent hepatic resection and colorectal resection for therapeutic purpose | Other malignancies, loss to follow-up or incomplete clinical data | NR |
| **Jin (2022)** | M1a: Metastasis limited to a single distant organ except for peritoneum | less than 20 years old, diagnosed with no positive histology and not only from a death certificate or autopsy, the information about the surgery to the primary site and metastatic lesion was missing, clinical pathological information (tumor size, carcinoembryonic antigen (CEA), T stage, N stage, histologic type, and neoadjuvant chemotherapy) was missing, survival information (survival month and final cause of death) was missing. | NR |

| | | | |
|---|---|---|---|
| **Zhai (2022)** | Curative-intent hepatic resection of CRLM | Patients who underwent ablation only, palliative liver resection (R2 resection), died perioperatively, had incomplete materials, or underwent combined hepatectomy and ablation for the tumour of maximum diameter, patients who received adjuvant chemotherapy before surgery. Among patients with extrahepatic disease during surgery, only patients who achieved an R0 resection of the extrahepatic disease were included in the study | 36.6 (IQR, 9.2–41.3) |
| **Liu (2021)** | Patients evaluated as having resectable CRLM at the initial diagnosis and before hepatic resection by a multidisciplinary team (MDT), metastasis diagnosed as liver-limited disease, preserved liver function and no other simultaneous malignancies. | Patients who had undergone only ablation or palliative hepatic resection (R2) | Development: 38, Validation: 34 |
| **Moro (2020)** | Patients who underwent resection of CRLM | Patients who underwent ablation only or palliative hepatectomy (R2 resection), and individuals with incomplete follow-up data | 27.6 (IQR: 14.0-47.6) |
| **Chen (2021)** | Pathologically proven colorectal adenocarcinoma liver metastases and treatment including NAC followed by hepatic resection for curative intent | Treatment with neoadjuvant radiotherapy, other malignancies and loss to follow-up or incomplete clinical data | NR |
| **Yao (2021)** | Histologically confirmed liver metastases of colorectal adenocarcinoma, patients underwent hepatic resection with simultaneous primary colorectal resection | Loss to follow-up or incomplete clinical data and other malignancies. | NR |
| **Kazi (2023)** | Patients with simultaneous operation for the CRLM and bowel cancer | Patients that were managed with thermal ablation for liver metastasis, those with CRLM requiring two-stage hepatectomy or preoperative volume augmentation for inadequate future liver remnant (FLR) and those with resections involving six or more liver segments | 42 (95% CI: 33.7–50.3) |

| | | | |
|---|---|---|---|
| **Meng (2021)** | Verified diagnosis of CRC by colonoscope and biopsy, synchronous liver metastasis demonstrated by enhanced computed tomography (CT) scan of abdomen or enhanced liver magnetic resonance (MR), R0 resection of primary lesion and liver metastasis, definite diagnosis of CRC with liver metastasis by postoperative biopsy, treated with postoperative systemic chemotherapy (5-FU-based) ± targeted therapy | Presence of extrahepatic metastasis, 30-day mortality from operative complications, loss to follow-up or absence of clinicopathological information | 26 |
| **Imai (2016)** | Patients with unresectable tumours assessed according to well defined selection criteria | - | 42.1 (range 5-251) |
| **Chen (2022)** | Histologically confirmed colorectal adenocarcinoma liver metastasis, received hepatectomy after NAC, developed recurrence. | Received neoadjuvant radiotherapy, loss to follow-up, missing clinical data. | NR |
| **Cheng (2022)** | Young-onset CRLM patients (aged 20–49 years, and CLRM evident at the initial diagnosis) | Patients diagnosed on the basis of autopsies or death certificates were excluded, as were those without comprehensive information | NR |
| **Kulik (2018)** | All consecutive primary liver resections for colorectal metastases | All cases with lack of sufficient follow-up data and all survivors with less than 10 years of follow-up | NR |
| **Bai (2021)** | Histologically confirmed colorectal adenocarcinoma, patients who underwent curative- intent CRLM resection, postoperative follow- up period of at least 3 months, and preoperative serum LDH values had to be available within 2 weeks before hepatectomy | Peritoneal metastasis, previous history of hepatectomy, R2 resection of liver metastases, ablation of metastatic sites or transcatheter hepatic arterial chemoembolization (TACE) within 4 weeks of study entry, patients in inflammatory conditions, and previous history of malignant tumor | 60.5 |
| **Wang (2021)** | Patients who underwent liver metastasectomy with curative intent and had adequate prehepatectomy serum samples and clinicopathological information | Patients with metastases in sites other than the liver or a history of prior hepatectomy, insufficient exosomal miRNA extraction, and substandard miRNA specimens were excluded | Development: 50.6, Validation: 32.7 |

| | | | |
|---|---|---|---|
| **Xu (2021)** | Pathologically confirmed CRLM patients after preoperative chemotherapy following liver resection | Patients who underwent direct surgery, underwent palliative surgery, having no pathologic response information, with missing clinical information, being lost to follow-up | NR |
| **Sasaki (2016)** | Patients who underwent curative-intent surgery for CRLM | Patients who only underwent ablation or patients who underwent a palliative liver resection (R2 resection). Among patients with extrahepatic disease at the time of hepatectomy, only patients in whom an R0 resection of the extrahepatic disease was achieved were included in the study population. Patients who underwent combined hepatectomy and ablation, who had ablation used for the tumor of maximum diameter were also excluded | 30.3 |
| **Wada (2022)** | Patients after hepatectomy for CRLM with gene expression profiling data | - | Training: 39.7 (95% CI: 34.5-44.4), Validation: 37.1 (95% CI: 26.9-47.8) |
| **Kim (2020)** | Patients who underwent hepatectomy for curative intent regardless of size, number, distribution while preserving a sufficient volume of functioning liver parenchyma more than 30% of whole liver without combining with radiofrequency ablation (RFA) and histologically proven colorectal adenocarcinoma liver metastasis | Extrahepatic metastases detected on preoperative work-up or during operation, in-hospital mortality within 30 days, R2 resection, noncancer-related mortality | Recurrence group: 63.2, non-recurrence group: 60.6 |
| **Dupre (2019)** | Patients who underwent their first curative-intent treatment with an open or a laparoscopic approach for CRLM | Unresectable extrahepatic disease (EHD) and/or if they underwent a two-stage hepatectomy and/or multiple primary colorectal cancer | Development: 50.5 (95% CI: 47.5-54.7) |
| **Qi (2023)** | Consecutive patients who underwent the initial and potentially curative resection for CRLM | Limited detectable continuous tumor area (<50 patches), patients with extrahepatic metastases, prior history of other malignancies or local treatment for liver metastases and patients without active postoperative follow-up | NR |

| | | | |
|---|---|---|---|
| **Wu (2021)** | The diagnosis of CRLM was confirmed through radiological examinations when the primary lesion was diagnosed by histopathology, the patients have received systemic chemotherapy before RFA, the response to pre-RFA chemotherapy was evaluated | Other treatment methods were received after ablation and before intrahepatic progression, patients who have no sufficient clinicopathological information and the evaluation of chemotherapy response, any other malignancy in the past 10 years, other factors affecting outcomes, such as clinical significant cardiovascular, uncontrolled hypertension, bleeding disorders or coagulopathy and active infection | NR |
| **Dasari (2023)** | Patients with at least two lesions on the anatomical right side and two lesions on the anatomical left side (bilobar). Liver surgery needed to be performed with curative intent with planned clearance of liver disease by any combination of surgical and ablative procedures | - | 50.9 |
| **Liu (2023)** | Expression profile information and complete survival data available | - | NR |
| **Amygdalos (2023)** | Consecutive adult patients undergoing elective liver resection in curative intent for CRLM | Emergency operations, resections of recurrent disease, or explorative laparotomies without resection, patients who died within the first 90 postoperative days and those who did not undergo complete resection of the primary tumor or liver metastases | Training: 51, Validation: 52 |
| **Chen (2023)** | Diagnosis of CRLM by ultrasound, CT, magnetic resonance imaging (MRI), or colorectal endoscopy, primary lesion of colorectal cancer having undergone radical resection, age ≥ 18 years old, no history of other tumors, no treatment received before admission | Receipt of emergency surgical treatment, no complete clinicopathological information, extrahepatic metastasis. | NR |
| **Wu (2018)** | Patients diagnosed with CRLM who underwent primary tumor resection | CRC was not diagnosed as the only primary cancer for the patients, patients were diagnosed via microscopic confirmation and not only from a death certificate or autopsy, patients were less than 18 years old and patients had missing or incomplete clinicopathological information | 14 (range: 0–71) |

| | | | |
|---|---|---|---|
| **Deng (2023)** | Clinical or histological evidence of CRLM, simultaneous resection of primary tumour and liver metastases with curative purpose and developed recurrence. | Loss to follow-up, incomplete clinical information and synchronous other malignant disease or infectious disease or other related diseases of liver and kidney | NR |
| **Berardi (2023)** | Patients who underwent upfront liver resection for resectable CRLM | Two-stage resections, patients who received systemic chemotherapy as neoadjuvant treatment were excluded, as were those who were treated exclusively with thermal ablation. Patients who did not receive adjuvant systemic treatment after liver resection were also excluded | NR |
| **Liu (2019)** | Patients received preoperative chemotherapy, tumour was considered to be resectable CRLM at final line of preoperative chemotherapy before hepatic resection by multidisciplinary team (MDT), preserved liver function (Indocyanine green, ICG<10%), all patients underwent hepatic resection with/without intraoperative radiofrequency ablation(RFA) aimed at achieving R0, no other simultaneous malignancies | Patients who underwent only ablation or palliative hepatic resection (R2) were excluded | 44 (range, 1-147) |
| **Welsh (2008)** | Consecutive patients undergoing primary or repeat hepatic resection for CRLM with curative intent | Patients with extra-hepatic disease | 26.4 (range: 0–211.2) |
| **Famularo (2023)** | First radiological diagnosis of CLM treated for the first time with liver resection, a feasible liver resection under a parenchyma sparing approach | Missing data on the follow-up variables, a progression-disease after neoadjuvant chemotherapy discovered at the radiological imaging (either CT or MRI scans) according to the RECIST criteria, and classic major hepatectomies as in case of right or extended right hepatectomy, left or extended left hepatectomy or trisectionectomy | 43 (IQR 22–66) |

| | | | |
|---|---|---|---|
| **He (2023)** | Histologically confirmed as colorectal cancer patients and presence of liver metastasis at the time of first diagnosis | Colorectal cancer is not the patient's first malignant tumor, patients with unknown surgical treatment information, patients with unknown differentiation degree, patients with unknown TNM stage, patients with unknown tumor diameter, and patients with unknown race | 22 |
| **Kattan (2008)** | Curative intent hepatic resection for CRLM, medical fitness for major laparotomy, no evidence of disseminated disease based on preoperative imaging and tumors confined anatomically within the liver to permit adequate liver parenchyma preservation. | - | NR |
| **Wensink (2023)** | Patients who underwent local treatment (resection and/or ablation) with curative intent for CRLMs | Patients with extrahepatic metastases before resection, R2 liver resections, appendiceal carcinoma, concomitant local liver treatment other than resection or ablation, and without any follow-up information | 35 |
| **Fendler (2015)** | CRC patients treated with SIRT, confirmed hepatic metastases from colorectal cancer, unresectable, progressive tumour refractory to chemotherapy, preserved liver function, as defined by a serum bilirubin≤2.0 mg/dl, performance status of functional impairment≥ 60 as measured with the Karnofsky index, pre-SIRT life expectancy of at least 3 months, fitness to undergo angiography | Liver failure according to the bilirubin threshold as defined above (>2.0 mg/dl) or by the presence of ascites, evidence of any uncorrectable hepatic arterial blood flow to the gastrointestinal tract observed at angiography or 99mTc-MAA (technetium-99- labelled macro-aggregated albumin) scintigraphy, pulmonary shunt exceeding 20 %, as estimated with 99mTc-MAA scintigraphy, or complete portal venous occlusion | Mean value: Training: 14 (range 2.3-55.5), Validation: 12.6 (range 2.1-48.8) |
| **Marfa (2016)** | Patients undergoing CRLM resection | Previous CRLM resection or refusal to participate in the study | NR |

| | | | |
|---|---|---|---|
| **Jiang (2023)** | Patients with resectable CRLM who underwent simultaneous resection of primary and hepatic lesions, neoadjuvant chemotherapy was not used | Incomplete clinical and pathological information (pathologic T [pT] stage, pathologic N [pN] stage, histology, chemotherapy record, survival month, and final cause of death), incomplete surgical resection of the primary and hepatic lesions (R2 resection), and extrahepatic metastasis | NR |
| **Endo (2023)** | Curative-intent hepatectomy for CRLM | Palliative or R2 resection, 2-stage hepatectomy (ie, planned staged hepatectomy), or previous hepatectomy for CRLM. Individuals with missing data on long-term outcomes and tumor characteristics (ie, tumor size and number) | 28.7 (IQR 13.7-52.0) |
| **Rees (2008)** | Primary or repeat hepatic resection for colorectal liver metastases with curative intent | - | 26.4 (range 0–211.3) |
| **Zakaria (2007)** | Patients who underwent resection of CRLM | Patients who had initial hepatic resection elsewhere or had only local ablative therapy | 36 (range, 0.2-444) |
| **Tan (2008)** | Patients who underwent resection of CRLM | Patients who had resections with positive margins of resection or who died within 30 days of operation, Patients who had resections with positive margins of resection or who died within 30 days of operation | 52.8 |
| **Hill (2012)** | Patients undergoing hepatic resection for metastatic colorectal adenocarcinoma | - | 50.1 |
| **Takeda (2021)** | Consecutive patients with a known RAS status who underwent initial hepatectomy with curative intent for CRLM | - | 29 |
| **Wang (2017)** | CRLM patients who received preoperative chemotherapy and underwent hepatic resection | - | 45 (range, 1–131) |

| | | | |
|---|---|---|---|
| **Spelt (2013)** | Consecutive cases of liver resection for CRLM | - | NR |

CRLM: Colorectal Liver Metastases, IQR: Interquartile Range, KRAS: Kirsten rat sarcoma virus, NR: Not Reported, NGS: Next Generation Sequencing, RFA: Radiofrequency Ablation, CRC: Colorectal Cancer, MDT: Multidisciplinary Team, BRAF: v-raf murine sarcoma viral oncogene homolog B1, CI: Confidence Interval, ALPPS: Associated liver partition with portal vein ligation for staged hepatectomy, NLR: Neutrophil-Lymphocyte Ratio, MWA: Microwave ablation, DEG: Differentially expressed genes, NAC: Neoadjuvant chemotherapy, LDH: Lactate dehydrogenase, SIRT: Selective Internal Radiotherapy

**Table S3:** Predictors included in clinical prediction models for prognosis of patients with colorectal liver metastases

| First Author (year) | Predictors |
|---|---|
| Buisman (2022) | Age, gender, location of primary, nodal status of primary, disease-free interval, number of CRLM, diameter of largest CRLM, preoperative CEA, resection margin, extrahepatic disease, KRAS mutation status, BRAF mutation status, histopathological growth pattern, perioperative systemic chemotherapy, and perioperative HAIP chemotherapy |
| Bertsimas (2022) | Age, gender, site, T and N stage of the primary tumour, size, distribution, and number of CRLM, presence of completely resected extrahepatic disease, margin width, use of intra-operative ablation |
| Bao (2021) | Gene mutations (BRCA2, LATS2, TSC2, KRAS, PIK3R1, PIK3CA), size of CRLM, lymph node status, primary site (right or left), number of metastases, CEA, metastasis status (synchronous or not) |
| Lam (2023) | Colorectal cancer nodal stage, neoadjuvant treatment, Charlson Comorbidity Score, pre-hepatectomy bilirubin, CEA, CRLM largest tumour diameter, extrahepatic metastasis, KRAS status |
| Reijonen (2023) | Node positive primary, Advanced T3-4 stage, multiple metastases, size >5cm, vitality >30%, margin <5mm, vascular invasion, biliary invasion |
| Margonis (2018) | Tumour lymph node status, CEA levels ≥ 20 mg/mL, extrahepatic disease, presence of KRAS mutation, TBS 3–8 and TBS ≥ 9 |
| Paredes (2020) | Age, sex, primary tumour location, T stage, receipt of chemotherapy before hepatectomy, lymph node metastases, number of metastatic lesions in the liver, size of the largest hepatic metastasis, CEA level and KRAS status. |
| Fruhling (2021) | Age, midgut location of primary, CRP, Albumin, Extended resection (more than 4 segments), number of CRLM |
| Taghavi (2021) | CEA level, adjuvant and neoadjuvant chemotherapy |
| Brudvik (2019) | LN positive primary, largest CRLM >5cm, RAS mutation status |
| Moaven (2023) | Initially 128 candidate predictors, GBT for OS included 59 variables, GBT for recurrence included 63 variables |
| Villard (2022) | Bilobar CRLM, extrahepatic disease, age < 60 years, number of CRLM, resectability, diameter of largest CLRM > 5cm, location of primary tumour |
| Chen (2020) | Modified TBS (number of tumours, maximum tumour diameter, bilobar disease), node positive primary, preoperative CEA >200 or CA19-9 >200, presence of KRAS/NRAS/BRAF mutations, extrahepatic disease |
| Chen (2022) | Model for 1 CRLM: CEA>200, CA 19-9 >50, preoperative pulmonary metastases, RAS mutation, right-sided primary / Model for 2-4 CRLM: primary T3-T4, primary LN positive, maximum tumour diameter > 5cm, preoperative pulmonary metastases, RAS mutation, preoperative CA 19-9 >50. / Model for >5 CRLM: primary LN positive, maximum tumour >5cm, RAS mutation |
| Dai (2021) | Number of CRLM >4, suspicion of positive LN, neurovascular invasion, location of primary, postoperative CEA >6, postoperative CA19-9 >13, Albumin >40 g/L |
| Liu (2021) | Early recurrence, LN positive primary, RAS mutation, no local treatment, size of CRLM >3cm |
| Liang (2021) | Largest size of CLM >5cm, site of recurrence (extra- or intrahepatic), CEA at recurrence, treatment of recurrence, time from hepatectomy to recurrence |

| | |
|---|---|
| Wu (2021) | Patients without neoadjuvant treatment CRLM >3, LN positive primary/ Patients with neoadjuvant therapy: number of CRLM >1, Transfusion during surgery |
| Sasaki (2022) | MCV, ALP, Albumin |
| Huiskens (2019) | Stage-1 risk score model: age > 67, baseline FLR to BW-ratio < 0.40, total center-volume < 20 cases / Stage-2 risk score model: age over 67, baseline FLR to BW-ratio below 0.40, bilirubin above 50 mmol/l 5 days after stage-1 and stage-1 morbidity of grade IIIa or higher (Clavien-Dindo) |
| Bai (2022) | LN positive primary, number of CRLMs >4, maximum diameter of CRLMs >4.4 cm, preoperative LDH levels > 250.5 U/L, and LHR (LDL/HDL ratio) > 2.9 |
| Fang (2022) | ApoA-I, NLR, cTN classification |
| Qin (2022) | Maximal size of CRLM, number of CRLMs, ablative margin, primary tumor lymph node status and chemotherapy |
| Kawaguchi (2021) | Maximal size of CRLM, number of CRLMs, RAS status |
| Zhang (2023) | 8 differentially expressed genes (DEGs) as a risk score / Full model: Age, M-stage, N-stage, T-stage, and risk score with DEGs |
| Chen (2021) | Complication model: preoperative serum GGT, preoperative RDW-CV and intraoperative blood loss / PFS model: preoperative GGT, D-Dimer, RDW-CV, RDW-SD, positive LN, bilobar disease, non-R0 resection. / OS model: preoperative GGT, primary LN metastases, non-R0 resection, major liver resection, complications, adjuvant chemotherapy |
| Jin (2022) | Age, T stage, N stage, Chemotherapy, Location of primary |
| Zhai (2022) | LN positive primary, preoperative CEA >200, interval between primary resection and diagnosis of liver metastases of < 12 months, TSR (Tumour Size Ratio) |
| Liu (2021) | LN positive primary, size of CRLM >5cm, right sided tumour, RAS mutation, multiple liver metastases |
| Moro (2020) | For wtKRAS patients: Primary LN positive, Number of CRLM >3, Size of CRLM >4,3cm / For mtKRAS patients: CEA, primary location (rectum or colon), sex |
| Chen (2021) | Complication model: preoperative GGT, major liver resection, intraoperative blood loss, right colon primary, primary LN positive / PFS model: Tumour regression grade (TRG), resection margin, NAC cycles >5, Primary LN positive / OS model: Resection margin, preoperative GGT, NAC toxicity, LN positive primary, NAC cycles >5 |
| Yao (2021) | Presence of LN metastases model: ASA score, preoperative albumin, differentiation, number of CRLM / PFS model: Differentiation, resection margin, bilobar disease, LN positive primary |
| Kazi (2023) | Comorbidities and major hepatectomy (defined as 4 or more segments) |
| Meng (2021) | LN positive primary, CEA, primary location, tumour grade (differentiation), number of CRLM |
| Imai (2016) | LN positive primary, number of CRLM, CA19-9, response to chemotherapy, extrahepatic disease |
| Chen (2022) | NAC cycles >4, preoperative creatinine, synchronous CRLM, postoperative complications |
| Cheng (2022) | CSS model: primary location, differentiation (grade), histologic type (mucinous or not), N stage, M stage, CEA, surgery (primary, metastatic or both) / OS model: primary location, differentiation (grade), N stage, M stage, surgery (primary, metastatic or both), metastases, CEA |
| Kulik (2018) | Model with preoperative variables: Age, Grade (differentiation), Chemotherapy, Hb, Quick / Model with pre- and perioperative variables: Age, margin in mm, chemotherapy, right liver resection, Quick, Hb, Grade (Differentiation), T stage |

| | |
|---|---|
| Bai (2021) | LDH-CRS model: LN positive primary, disease- free interval from the diagnosis of primary tumour <12 months, number of CRLM >1, maximum CRLM diameter >5 cm, preoperative CEA levels >200 ng/ml, and preoperative LDH levels >ULN / m-CRS model: LN positive primary, disease- free interval from the diagnosis of primary tumour <12 months, number of CRLM >1, maximum CRLM diameter >5 cm, and preoperative LDH levels >ULN |
| Wang (2021) | mRNA risk score: 4 circulating exosomal microRNAs / merged risk score: circulating exosomal miRNA risk score, Fong score |
| Xu (2021) | Disease-free interval <12 months, tumour size, tumour number, RAS status |
| Sasaki (2016) | TBS: maximum tumour diameter, number of CRLM |
| Wada (2022) | Transcriptomic panel with 6 genes (COX6A1, ERN1, IFITM2, S100P, STK24, and TMTC3) / Mixed model: tumour number, synchronous lesions, CA19-9, and CEA, transcriptomic panel |
| Kim (2020) | Synchronicity, CA19-9, Number of CRLM, largest CRLM size, resection margin, NLR, PNI |
| Dupre (2019) | Preoperative model: ASA score, location of the primary tumour, LN positive primary, number and size of CRLM, NLR / Postoperative model: same plus resection margin |
| Qi (2023) | Risk score with 4 SOFs (histological features) / Combined SOF-CRS score with addition of Fong score |
| Wu (2021) | LN positive primary, CR LM number, CRLM size, chemotherapy response, KRAS status |
| Dasari (2023) | Age, ASA, primary location, N stage, Synchronicity, CRLM number, resection type (one-stage or two-stage), margin status, adjuvant chemotherapy, major complications, chemotherapy response |
| Liu (2023) | Risk score with 7 differentially expressed genes (DEGs): HAMP, COLEC11, MMP3, UGT2B7, C8G, SERPINA1, IFITM10, Age, and AJCC stage |
| Amygdalos (2023) | Preoperative CEA, age, diameter of the largest CRLM, number of CRLM, BMI, and primary tumour differentiation |
| Chen (2023) | CA19-9, N stage, Ki-67, LVI, pMMR, number of CRLMs |
| Wu (2018) | OS model: Age, marital status, primary location, grade (Differentiation), N stage, tumour size, CEA, tumour deposits, circumferential resection margin/ CSS model: same |
| Deng (2023) | LN positive primary, postoperative complications, NLR, BUN, TBS (Tumour burden score) |
| Berardi (2023) | Primary location, interval from primary to CRLM, CRLM number, CRLM size |
| Liu (2019) | LN positive primary, CRLM size >5cm, increase in tumour diameter during first-line chemotherapy, RAS mutation, multiple CRLMs |
| Welsh (2008) | Non-anatomical resection, more than three hepatic metastases involving >50% of the liver, repeat hepatic resection, bilobar metastases, abnormal pre-operative LFTs |
| Famularo (2023) | Planned R1 vascular resection, number of CRLMs, primary location, CEA, sex |
| He (2023) | CEA, chemotherapy, N stage, grade (Differentiation), lung metastases |
| Kattan (2008) | Gender, age, primary location, disease-free interval, preop CEA, CRLM number, CRLM largest size, bilateral resection, bilobar disease, N stage |
| Wensink (2023) | Location of the primary tumour, T stage, N stage, RAS/BRAFV600E mutational status, and number and size of CRLMs |
| Fendler (2015) | No prior liver surgery, CEA, transaminase toxicity ≥2.5× ULN, CRLM size ≥10 cm |
| Marfa (2016) | Tissue proteomic profile with 4 protein peaks |
| Jiang (2023) | OS model: CEA, N stage, adjuvant chemotherapy / CSS model: same |

| | |
|---|---|
| Endo (2023) | OS-OPT model: T stage of the primary tumour, primary tumour location, KRAS status, TBS, CEA, neoadjuvant treatment, lymph node metastases of the primary tumour, synchronous CRLM, and Charlson comorbidity score / RFS-OPT model: KRAS status, TBS, primary tumour lymph node metastases, neoadjuvant treatment, synchronous CRLM, Charlson comorbidity score |
| Rees (2008) | Preoperative score: LN positive primary, grade (Differentiation), CEA, CRLM number, CRLM size, extrahepatic disease / Postoperative score: LN positive primary, grade (Differentiation), CEA, CRLM size, positive resection margin, extrahepatic disease |
| Zakaria (2007) | Liver metastasis in <30 months, CRLM size >8 cm, blood transfusion, positive hepatoduodenal nodes |
| Tan (2008) | Tumour grade (Differentiation), LN positive primary |
| Hill (2012) | CEA >200 ng/ml, recurrence pattern (liver only or lung only, extrahepatic or liver and lung, liver and extrahepatic) |
| Takeda (2021) | Number of CRLM >4, CA19-9 > 100 U/mL at hepatectomy, presence of mutant RAS |
| Wang (2017) | Poor preoperative chemotherapy response, Fong clinical risk score > 2, KRAS mutation |
| Spelt (2013) | ANN model (top-6 variables): age, administration of preoperative chemotherapy, size of the largest CRLM, occurrence of haemorrhagic complications, preoperative CEA-level, number of CRLM / Cox model: age, ASA-score III or IV, bleeding during operation, biliary complications, size of the largest CRLM, administration of preoperative chemotherapy |

CRLM: Colorectal liver metastases, KRAS: Kirsten rat sarcoma virus, BRAF: v-raf murine sarcoma viral oncogene homolog B1, HAIP: Hepatic arterial infusion pump, TBS: Tumour burden score, CRP: C-reactive protein, LN: Lymph node, GBT: Gradient-boosted trees, CA19-9: Carbohydrate antigen 19-9, MCV: Mean corpuscular volume, ALP: Alkaline phosphatase, FLR: Future liver remnant, BW: Body weight, LDH: Lactate dehydrogenase, ApoA-I: Apolipoprotein AI, NLR: Neutrophil-to-lymphocyte ratio, DEGs: Differentially expressed genes, GGT: Gamma-Glutamyl Transferase, RDW-CV: Red cell distribution width - coefficient of variation, RDW-SD: Red cell distribution width - standard deviation, PFS: Progression-free survival, NAC: Neoadjuvant chemotherapy, ASA: American Society of Anesthesiologists. Hb: Hemoglobin, CSS: Cancer-specific survival, CRS: Clinical risk score, ULN: Upper limit normal, PNI: Prognostic nutrition index, SOFs: Spatial organization features, CEA: Carcinoembryonic antigen, BMI: Body mass index, pMMR: Mismatch repair proficiency, LVI: Lymphovascular invasion, CBC: Complete blood count, LFTs: Liver function tests, OPT: Optimal policy tree, BUN: Blood urea nitrogen, ANN: Artificial neural network
